# Intramural Intestinal Metastasis of Malignant Melanoma as a Rare Cause of Ileoileal Intussusception: A Case Report and Review of the Literature

**DOI:** 10.1155/cris/5556332

**Published:** 2025-01-16

**Authors:** Gary Amseian, Alexandre Soler, Alba Torroella, Lia Sisuashvili, Paula Escarcena, Gerard Rafart

**Affiliations:** ^1^Department of Radiology, Hospital Clínic de Barcelona, Barcelona, Spain; ^2^Department of General and Digestive Surgery, Hospital Clínic de Barcelona, Barcelona, Spain; ^3^Department of Pathology, Hospital Clínic de Barcelona, Barcelona, Spain

**Keywords:** case report, emergency radiology, emergency surgery, intussusception, metastatic melanoma

## Abstract

Intussusception in adults is rare and poses a diagnostic challenge, often due to neoplastic causes. Metastatic melanoma is known to spread to the gastrointestinal tract, especially the small intestine. We report the case of a patient with obstructive symptoms and a history of metastatic melanoma. An emergency abdominal computed tomography (CT) scan identified an obstruction caused by ileoileal intussusception located at the site of a previously described enteric metastasis. Following palliative surgery with ileal resection, two nodular lesions causing intussusception were identified, and pathological examination confirmed intramural metastases from melanoma. In adult patients with obstructive symptoms and a history of melanoma, intussusception secondary to intestinal metastases should be considered in the differential diagnosis.

## 1. Introduction

The differential diagnosis of small bowel obstruction includes adhesions, hernias, malignancy, volvulus, and inflammatory bowel disease but is not limited to these entities [1]. Intussusception, or invagination, involves the telescoping of a proximal segment of the intestine into the lumen of an adjacent segment, leading to obstruction. Over 95% of intussusception cases are diagnosed in the pediatric age group [2]. The majority of pediatric cases are idiopathic and ileocolic in location [3], though intussusception in pediatric patients can also occur in the small intestine and be secondary to benign causes like heterotopic pancreas [4] or malignant ones [5].

In contrast, intussusception in adults is a rare occurrence, typically secondary to a neoplastic origin [6]. When affecting the small intestine, these neoplasms are usually benign (like lipomas or hamartomatous polyps), although there are other less common origins such as malignant lesions [7]. Malignant melanoma is a neoplasm that forms in areas containing melanocytes, such as the skin, eyes, meninges, and anal region. It can also originate as a primary tumor in the small intestine and colorectal region [8], but primary intestinal melanomas are uncommon [9]. In a study that investigated the metastatic pattern of 216 patients with histologically confirmed advanced malignant melanoma, and in whom metastases were documented through autopsy in macroscopic and microscopic examinations, peritoneal metastases were observed in approximately 43% of the cases and small intestine metastases in approximately 36% of the cases [10]. Due to this propensity of melanoma to metastasize to the gastrointestinal tract and peritoneal surfaces, most cases of melanoma in this location result from metastasis from skin melanoma [11]. The tendency of metastatic melanoma to initiate intussusception by forming a gastrointestinal mass has been documented in a few cases and is considered a rare manifestation [11].

We present a rare case of ileoileal intussusception secondary to intramural implants of malignant melanoma. One of the peculiarities of this case is that these implants were recorded through abdominal imaging prior to the acute presentation of intussusception and were later confirmed surgically by laparotomy.

## 2. Case Presentation

We present the case of a patient with a history of multiple melanomas, treated with surgical excision, but with no history of abdominal surgery. After surgical excision of local skin lesions, the patient continued with clinical and analytical follow-up through annual controls of S100 protein and also with triennial plain chest radiographs, which did not reveal any significant abnormalities. Eleven years after surgical skin excision, an elevated level of protein S100 during follow-up prompted a thoracoabdominal computed tomography (CT) scan that revealed metastatic progression with multiple visceral metastases, including enteric and mesenteric implants. Brain magnetic resonance imaging revealed CNS dissemination, and the cytological study of lymph nodes confirmed a stage IV BRAF-mutated malignant melanoma. Consequently, systemic treatment with BRAF inhibitors was initiated.

**Figure 1:**
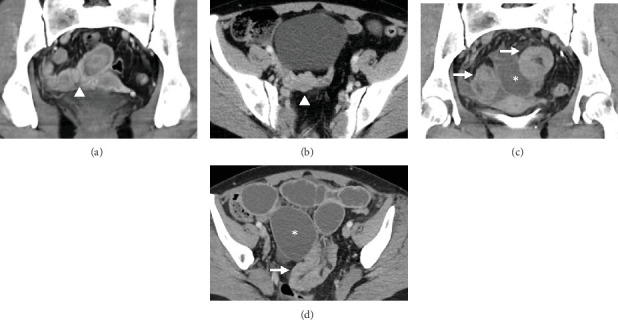
Ileoileal intussusception secondary to intestinal metastasis on abdominal computed tomography (CT). In light of the suspicion of progression of the metastatic melanoma disease, a whole-body CT scan was conducted, which confirmed progression. Coronal (a) and axial (b) slices of abdominal CT revealed multiple nodules and focal parietal thickening of the intestinal loops (arrowheads in a and b) suggestive of intramural implants. Months after that last CT, the patient presented at the emergency department with obstructive symptoms, so a second abdominopelvic CT scan was conducted. Coronal (c) and axial (d) slices showed distension of jejunal and ileal loops up to a caliber of 5 cm (asterisks in c and d) proximal to the insertion of a 10-cm-long segment of the distal ileum into the contiguous ileal loop (arrows in c and d), at the site of one of the enteric metastatic implants described in the previous CT. This was interpreted as intestinal obstruction secondary to ileoileal intussusception, probably in relation to intestinal implants of metastatic melanoma.

In subsequent months, the patient experienced several episodes of vomiting, nausea, and abdominal pain. CT scans that monitored the patient's condition showed a partial radiological response. After discontinuing medical treatment, digestive symptoms intensified, and the patient presented to the emergency department with obstruction, which led to an emergency abdominal CT scan. The images showed marked distension of the jejunum and ileum proximally to a sudden change in caliber due to a telescoping of distal ileal loops, which was topographically aligned with one of the enteric implants observed in a previous abdominal CT scan conducted 9 months earlier (Figure 1).

**Figure 2:**
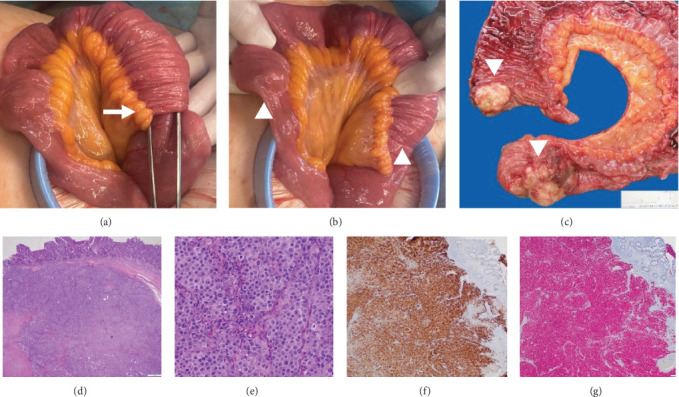
Laparotomy with ileotomy in intramural melanoma metastases. After identifying intestinal obstruction secondary to intussusception on computed tomography (CT), the patient was considered for palliative surgery. During laparotomy, a 10-cm ileoileal intussusception (arrow in a) with retrograde distension of intestinal loops was observed. After the intussusception was reduced, two nodular lesions were observed inside the terminal ileum (arrowheads in b), which had led to the telescoping of ileal loops. Macroscopic examination of the surgical ileotomy specimen identified two solid nodular exophytic tumors in the resected ileal segment (arrowheads in c). Microscopic examination (d and e, hematoxylin and eosin staining) revealed the infiltration of the ileal wall by two tumors with a solid pattern and areas of necrosis, cells of epithelioid appearance with broad eosinophilic cytoplasm, atypical nuclei and prominent nucleoli, and the presence of mitotic figures. The immunohistochemical examination was positive for Melan A (f) and SOX10 (g), all of which was conclusive for intramural melanoma metastases.

The findings were interpreted as intestinal obstruction due to invagination of the distal ileum, likely resulting from intestinal metastases. As a result, the patient was considered for a palliative surgical intervention. During laparoscopic surgery, a 10-cm intussusception was identified in the distal ileum (Figure 2a), accompanied by retrograde intestinal distension. Following surgical reduction, two nodular lesions were identified in the terminal ileum (Figure 2b), which caused the insertion of one intestinal segment into the adjoining loop. Given the proximity of the lesions, a single ileal resection was performed, followed by a side-to-side anastomosis. Macroscopic examination of the surgical specimen identified two solid nodular exophytic tumors in the resected ileal segment (Figure 2c), one of them extending to the serosa. Microscopic examination (Figure 2d–g) revealed their neoplastic nature and positivity to Melan A and SOX10 in the immunohistochemical examination, all of which was conclusive for intramural intestinal metastases of melanoma.

## 3. Discussion

In a patient with a history of malignant melanoma with gastrointestinal carcinomatous involvement and without hernias or a history of abdominal surgery, as in the case previously described, the primary diagnostic consideration for intestinal obstruction should be a peritoneal implant even if it had not been detected in prior CT scans. Despite its rarity, intussusception caused by intramural intestinal metastases should be taken into account in adult patients presenting with obstructive symptoms and a history of malignant melanoma during the differential diagnosis process. In this scenario, CT with intravenous contrast is the imaging modality of choice [12], although on many occasions it is not possible to assess the presence of peritoneal implants due to the low diagnostic sensitivity of this technique for peritoneal carcinomatosis, and in those cases, the diagnosis is made during the surgical procedure itself.

Other publications have reported cases of intestinal intussusception secondary to metastatic implants of malignant melanoma frequently at the gastrointestinal or at the small bowel level [9, 11, 13, 14]. However, one of the unique aspects of the case we present lies in having been able to record the presence of intramural intestinal metastatic implants through abdominal radiological imaging prior to the acute presentation as intussusception. To the best of our knowledge, this feature had not been documented in other cases reported in literature. The patient presented clinical signs of intestinal obstruction and, on the contrast-enhanced CT (CECT), an intussusception secondary to the already known intestinal metastasis was clearly evident, which was later confirmed during the surgical procedure.

Intussusception in adults poses a diagnostic challenge due to its variable presentation, rarity, and low index of suspicion [15]. In the context of melanoma, the estimated time between the diagnosis of the primary lesion and the appearance of metastasis in the digestive tract averages 4.5 years [14], further complicating diagnostic suspicion. A range of systemic therapeutic agents is currently available for patients with intestinal metastatic melanoma, including chemotherapy, BRAF-targeted therapies, and immune checkpoint inhibitors. Surgery remains, though, the treatment of choice, acting more as an effective palliative treatment rather than a cure [16]. Broad resection of lesions with adequate margins and accompanying mesentery wedge for lymph node removal is the preferred approach [17].

In these patients, the role of abdominal imaging studies is pivotal, and complete surgical resection of the lesion can be generally performed safely, providing symptom control and improved survival [18].

## Data Availability

The data and materials generated and/or analyzed during the current study are available from the corresponding author on reasonable request.

## Nomenclature

CECT: Contrast-enhanced computed tomography.
